# Durability of heat-treated *Paulownia tomentosa* and *Pinus koraiensis* woods in palm oil and air against brown- and white-rot fungi

**DOI:** 10.1038/s41598-023-48971-z

**Published:** 2023-12-11

**Authors:** Intan Fajar Suri, Byantara Darsan Purusatama, Jong Ho Kim, Wahyu Hidayat, Won Joung Hwang, Apri Heri Iswanto, Se Yeong Park, Seung Hwan Lee, Nam Hun Kim

**Affiliations:** 1 Department of Forest Biomaterials Engineering, College of Forest and Environmental Sciences, Kangwon National University, Chuncheon, 24341 Republic of Korea; 2 Institute of Forest Science, Kangwon National University, Chuncheon, 24341 Republic of Korea; 3 Department of Forestry, Faculty of Agriculture, University of Lampung, Bandar Lampung, 35145 Indonesia; 4 Wood Engineering Division, Forest Products and Industry Department, National Institute of Forest Science, Seoul, 02455 Republic of Korea; 5 Department of Forest Products Technology, Faculty of Forestry, Universitas Sumatera Utara, Deli Serdang, 20353 North Sumatra Indonesia

**Keywords:** Engineering, Materials science

## Abstract

This study aimed to evaluate and compare the effects of oil- and air-heat treatments on the durability of *Paulownia tomentosa* and *Pinus koraiensis* woods against *Fomitopsis palustris* and *Trametes versicolor*. The wood samples were treated in palm oil and air at 180, 200, and 220 °C for 2 h. The weight loss, morphology, crystalline properties, and chemical compounds of untreated and heat-treated wood after fungal attack were investigated. The significant difference in weight loss between oil- and air-heat-treated samples was shown at 220 °C. Heat-treated wood exposed to white-rot fungus showed a lower weight loss than that exposed to brown-rot fungus. The cell components in the untreated- and heat-treated *Paulownia tomentosa* and *Pinus koraiensis* at 180 °C were severely damaged due to fungal exposure compared to those at 220 °C. A fungal effect on the relative crystallinity was observed in heat-treated wood at 180 °C, whereas the effect was not observed at 220 °C. Following brown-rot fungus exposure, untreated- and heat-treated wood at 180 °C showed a notable change in the Fourier transform infrared (FTIR) peaks of polysaccharides, whereas no noticeable change in lignin peaks was observed. Heat-treated wood at 220 °C showed no noticeable change in the FTIR spectra owing to brown-rot fungus exposure. Exposure to white-rot fungus did not noticeably change the FTIR spectra of untreated and heat-treated wood.

## Introduction

Domestic commercial wood species, such as *Paulownia tomentosa* and *Pinus koraiensis,* are important timber resources in Korea. The *Paulownia* tree is a fast-growing species with excellent characteristics for timber resources, showing clear wood grains, high dimensional stability, light weight, high strength-to-weight ratio, and high durability^1,2^. Paulownia wood is generally used in construction, furniture, pulp, handicrafts, particleboards, paper production, bioenergy applications, and musical instruments^2–6^.

*Pinus koraiensis* is widely planted across the northern part of South Korea and is an important raw material for commercial use in the Korean wood industry^7^. The wood has a high growth rate and low shrinkage, making it suitable for use in construction, furniture, particleboard, fiberboard, and wooden ships^2,8,9^. However, *Pinus koraiensis* wood has lower density and natural durability concerning wood fungi compared to other commercial pine woods, such as *Pinus densiflora* and *Pinus thunbergii*^2^. In addition, *Paulownia tomentosa* and *Pinus koraeinsis* woods have a light creamy or pale brown color that is not an aesthetic advantage for some applications.

Heat treatments with various heating media, such as air, nitrogen, steam, oil, and vacuum have been used to improve wood properties, such as dimensional stability, hydrophilicity, color, and durability^10–18^. Air heat treatment (AHT) is a cost-effective and simple method for wood modification by heat treatment^12,14^, and recently, oil heat treatment using edible oils, such as palm and vegetable oil, is also a reasonable method for improving wood properties through the synergetic effects of oil and heat^19–23^.

Recently, we studied the effects of heat treatment on the physical and mechanical properties of *Paulownia tomentosa* and *Pinus koraiensis* woods^20–22,24–26^. Hidayat et al.^24,25^ reported that the equilibrium moisture content and wettability of *Paulownia tomentosa* and *Pinus koraiensis* woods heat-treated in air at 160, 180, 200, and 220 °C for 2 h decreased with increasing temperature. In addition, the color of the wood darkened as temperature increased, which was preferred by consumers compared to the lighter colors of the untreated wood. Kim et al.^26^ also reported that the lightness (L*) and weight of the *Paulownia tomentosa* heat-treated in air at 160, 180, 200, and 220 °C for 2 h decreased with increasing temperature, while the density and relative crystallinity were constant and increased, respectively*.* Suri et al.^20–22^ reported that oil-heat-treated (OHT) specimens showed a darker color, lower volume shrinkage, lower weight loss in abrasion, higher density, higher compressive strength, and higher hardness than air-heat-treated specimens. In addition, heat treatment with oil increased the weight of the samples owing to oil uptake by the wood sample, whereas the air heat treatment tended to decrease the sample weight.

Fungal decay caused by brown-, white-, and soft-rot fungi is the most (important) and widespread wood degradation type. Brown- and white-rot are the two major types of wood decay caused by *Basidiomycetes*. Brown-rot fungi primarily degrade carbohydrates and preferentially amorphous components, whereas white-rot fungi degrade all structural cell-wall components^27^. Heat treatment using air^28–30^ and oil^11,16^ has been used to improve the durability of wood against brown- and white-rot fungi.

As previously described^20–22^, oil-heat-treatment is a more effective method for improving the physical and mechanical properties of *Paulownia tomentosa* and *Pinus koraiensis* woods than AHT. However, there are insufficient comparative studies on the fungal resistance properties of air- and palm oil-heat-treated *Paulownia tomentosa* and *Pinus koraiensis* woods. Therefore, in the present study, the durability of oil-heat-treated (OHT) and air-heat-treated (AHT) *Paulownia tomentosa* and *Pinus koraiensis* woods was investigated to provide information regarding the effective utilization of both wood species.

## Materials and methods

### Materials

The information on the sample trees used in the present study is the same as our previous studies^20–22^. Three trees each of 15–20 years old *Paulownia tomentosa* and 30–35 years old *Pinus koraiensis* were obtained from the research forest of Kangwon National University, Chuncheon, Korea (37° 77′ N, 127° 81′ E). All trees were obtained in compliance with all institutional, national, and international guidelines and legislation. In addition, permission to collect all sample trees was obtained from the dean of the College of Forest and Environmental Sciences, Kangwon National University. Basic information on the tree samples is listed in Table 1. Defect-free quarter-sawn boards with dimensions of 200 mm (longitudinal) × 90 mm (radial) × 20 mm (tangential) were prepared from near the bark. The samples were oven-dried and conditioned in a desiccator at 25 ± 5 °C under a relative humidity of 65% until reaching an equilibrium moisture content of 10%. Brown- (*Fomitopsis palustris* FRI 21055) and white-rot (*Trametes versicolor* FRI 20251) fungi were obtained from the National Institute of Forest Science of the Korea Forest Service, Seoul, Korea, and were used for the fungal resistance test.

**Table 1:** Basic information of the sample trees.

Common name	Scientific name	Age (years)	D.B.H (mm)	Air-dried density (g/cm^3^)	Location
Royal paulownia	*Paulownia tomentosa* (Thunb.) Steud	15–20	280–330	0.30 (0.05)	Chuncheon, Korea (37°77′ N, 127°81′ E)
Korean white pine	*Pinus koraiensis* Siebold & Zucc	30–35	280–320	0.42 (0.05)	

*Numbers in parentheses are standard deviations.

### Heat treatment

In the present study, the oil and air heat-treatment were performed according to the previous study^20–22^. A lab-scale oil bath with a volume of 5 L (C-WHT-S2, Chang Shin Science, Seoul, Korea) was used for the heat treatment of the wood, and commercial palm oil (Lotte Foods, Korea) was used as the heating medium. Wood samples with the dimension of 200 mm (longitudinal) × 90 mm (radial) × 20 mm (tangential) were fully soaked in the oil bath and the temperature was then raised from a room temperature of 25 ± 5 °C to target temperatures of 180, 200, and 220 °C at a heating rate of 2 °C/min. The target temperature was maintained for 2 h, and the samples were removed from the oil bath after the bath reached the room temperature of 25 ± 5 °C. Finally, the OHT samples were drained at 27.5 ± 3 °C and RH 60 ± 5% for 24 h.

The AHT of the wood samples was performed in an electric furnace (Supertherm HT 16/16; Nabertherm GmbH, Lilienthal, Germany). The temperature was raised from a room temperature of 25 ± 5 °C to the target temperatures of 180, 200, and 220 °C with a heating rate of 2 °C/min, and the heat-treated samples were removed from the furnace chamber after cooling.

Wood samples treated in air and palm oil were oven-dried at 103 ± 3 °C for 24 h and kept in a desiccator with silica gel for one week.

### Fungal resistance test

Untreated and heat-treated wood samples with dimensions of 20 (L) mm × 20 (R) mm × 20 (T) mm were used for the fungal resistance test, and detailed information on the samples is summarized in Table 2. The wood samples were sterilized in an autoclave at 121 ± 1 °C after measuring the initial oven-dried weights. Fungal resistance tests were performed according to KS F 2213 (2018) using brown- (*Fomitopsis palustris*) and white-rot (*Trametes versicolor*) fungi.

**Table 2:** Sample information for evaluating the durability of heat-treated woods.

Test	Sample dimension	Species	Heating media	Temperature and duration	Sample number*	Total
Fungal resistance test (KS F 2213 (2018))	20 (L) × 20 (R) × 20 (T) mm^3^	*Paulownia tomentosa* and *Pinus koraiensis*	Oil and air	Control 180 °C for 2 h 220 °C for 2 h	3 × 2 3 × 2 × 2 3 × 2 × 2	30

*Sample number = Replication × species × heating media.

A 900 mL glass bottle was filled with 250 g of air-dried sea sand sieved between 10 and 20 mesh screens with 3 g of peptone, 15 g of malt extract broth, and 40 g of dextrose, sterilized at 121 ± 1 °C for 15 min, and inoculated with fungi. After cultivating sufficient inoculated fungi, the sterilized untreated and heat-treated samples were placed in bottles, with three samples in each bottle. The fungi were then incubated for eight weeks (56 d) under a relative humidity of 70 ± 2% and a temperature of 27 ± 2 °C, separately. Thereafter, fungal hyphae from the decayed wood surfaces were removed using a brush. The fungal resistance tested wood samples were air-dried for 24 h and then oven-dried at 60 ± 2 °C for 48 h before weighing the samples.

### Weight loss and morphology observation

To determine the decay resistance of untreated and heat-treated wood samples, the weight loss (WL) of the samples was measured using Eq. (1).

1 $$WL \, (\% ) \, = \, (W_{B} - W_{A} )/W_{B} \times 100$$

where *W*_*B*_ and *W*_*A*_ represent the weight of the samples before and after the fungal attack (g), respectively. The resistance levels of each wood sample were classified according to the Korean standard (KS F 2213 (2018)), as listed in Table 3.

**Table 3:** Weathering classification according to the Korean standard (KS F 2213 (2018)).

Average weight loss (%)	Classification
0–10	Highly resistant
11–24	Resistant
25–44	Moderately resistant
≥ 45	Slightly or non-resistant

The macroscopic characteristics of the cross-sections of the decayed samples were visually observed. Photographs were captured using a mobile phone (Samsung Note 10, 12MP with F1.8, Suwon-si, Korea).

Oven-dried samples with dimensions 5 mm (L) × 5 mm (R) × 5 mm (T) were coated with gold using a Cressington sputter coater (ULVAC G-50DA, Japan). The transverse surfaces of the wood samples, before and after fungal exposure, were observed using a scanning electron microscope (JSM-5510; JEOL, Japan, 15 kV).

### Crystalline characteristics

The crystalline properties such as the relative crystallinity (RC) and crystallite width (CW) of the samples before and after the fungal resistance test were measured using an X-ray diffractometer (DMAX2100V; Rigaku, Tokyo, Japan, Cu target, 40 kV, 30 mA). Three samples of approximately 1 mm thickness (R), 15 mm width (T), and 15 mm length (L) were prepared from each sample before and after the decay resistance test. RC and CW were measured using Segal’s^31^ and Scherrer’s methods^32^, as shown in Eqs. (2) and (3), respectively.

2 $$RC{ (}\% {)} = ((I_{200} - I_{am} )/I_{200} ) \times 100$$

where *I*_*200*_ and *I*_*am*_ represent the diffraction intensities of the crystalline region at 2θ = 22.8° and the amorphous region at 2θ = 18°, respectively.

3 $${\text{CW }}({\text{nm}}) = {\text{K}}\lambda /\beta {\text{cos}}\theta$$

where CW, *K*, and *λ* represent the crystallite width, Scherrer constant (0.9), and the X-ray wavelength (λ = 0.1542 nm), respectively. *β* and *θ* denote the half-width in radians and the Bragg angle, respectively.

### Chemical composition analysis

The chemical composition of untreated and heat-treated samples before and after the decay resistance test was examined using the attenuated total reflection method in the range of 400–4000 cm^−1^ with an FT-IR spectrometer (Nicolet Summit, Thermofisher scientific, USA), installed at the Department of Forest Biomaterials Engineering at Kangwon National University.

### Statistical analysis

Statistical differences in weight loss between the samples after the fungal resistance test were analyzed using univariate analysis of variance and post-hoc Duncan’s multiple range tests using IBM SPSS software (SPSS ver. 26, IBM Corp., New York, USA).

### Ethical approval

This article does not contain any studies with human participants or animals performed by any of the authors.

## Results and discussion

### Weight loss

**Figure 1:**
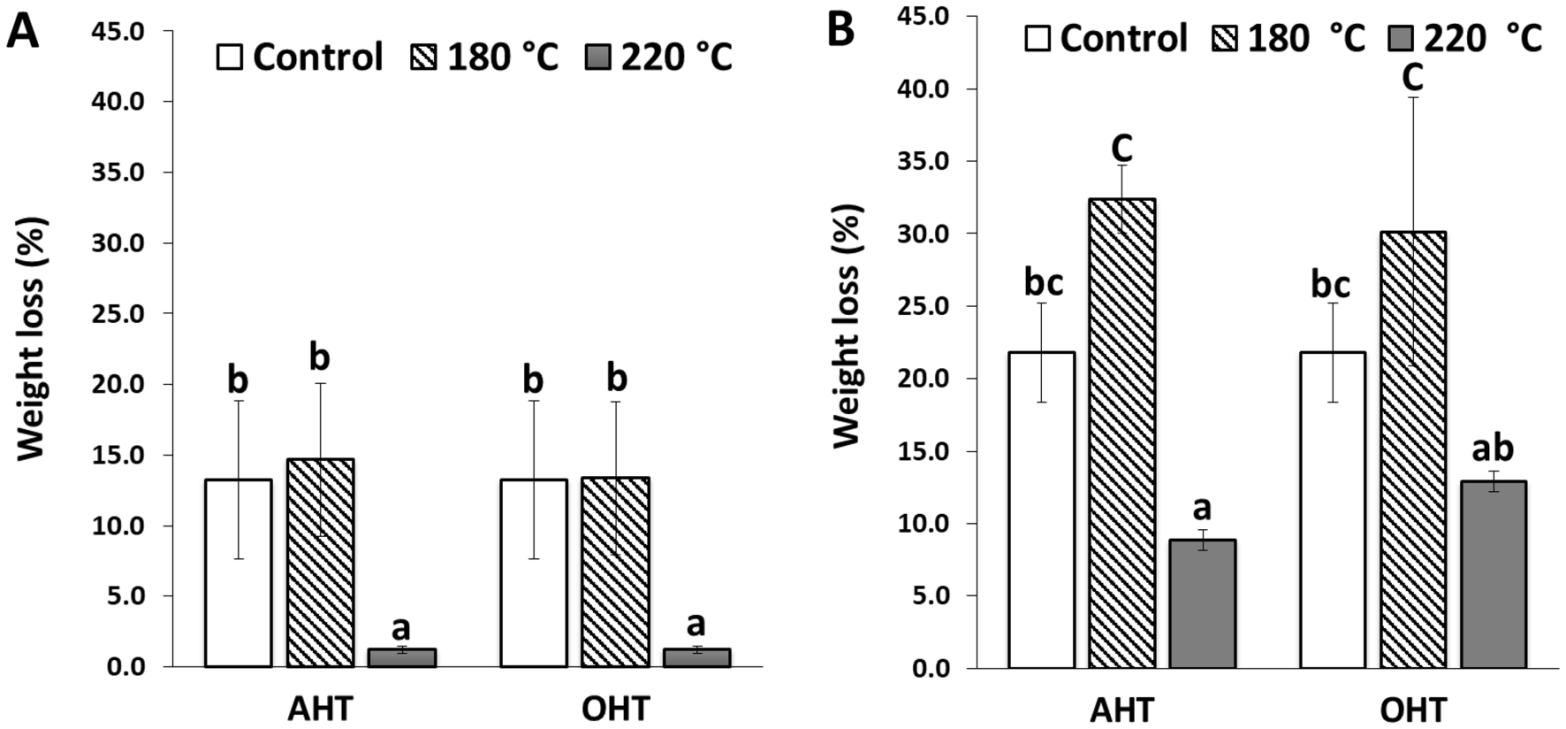
Weight loss of the untreated and heat-treated *P. tomentosa* (**A**) and *P. koraiensis* woods (**B**) exposure to *Fomitopsis palustris*. The same lowercase letters above the mean values in the histograms denote insignificant outcomes between the samples at a 5% level of significance based on Duncan’s multiple range test.

Figure 1 shows the weight loss after being exposed to brown-rot fungus of the heat-treated *P. tomentosa* and *P. koraiensis* wood in oil and air.

After exposure to brown-rot fungus, the untreated *P. tomentosa* wood lost 13.4% in weight. The weight loss of oil- and air-heat-treated wood at 180 °C was 13.4% and 14.7%, respectively, which were not significantly different from the untreated wood. This tendency indicated that the untreated and heat-treated woods in oil and air at 180 °C are classified as resistant (Table 3). In addition, the *Paulownia* wood heat-treated in oil and air at 220 °C showed the lowest weight loss of 1.2%, which was significantly different from the untreated and heat-treated wood at 180 °C. Both heat treatments in oil and air at 220 °C improved the fungal resistance of *Paulownia* wood to highly resistant. Furthermore, no significant difference in the weight loss between the oil- and air-heat-treated wood samples at each temperature was noted.

In *P. koraiensis* wood*,* the weight loss was 21.8% for untreated wood, 30.1% for OHT wood at 180 °C, and 32.4% for AHT wood at 180 °C, which are classified as moderately resistant. The heat-treated wood at 180 °C showed significantly higher weight loss than untreated wood. The weight loss significantly decreased in the OHT and AHT wood at 220 °C, at 12.9% and 8.8%, respectively. Moreover, there was a significant difference in weight loss between OHT and AHT samples at 220 °C.

**Figure 2:**
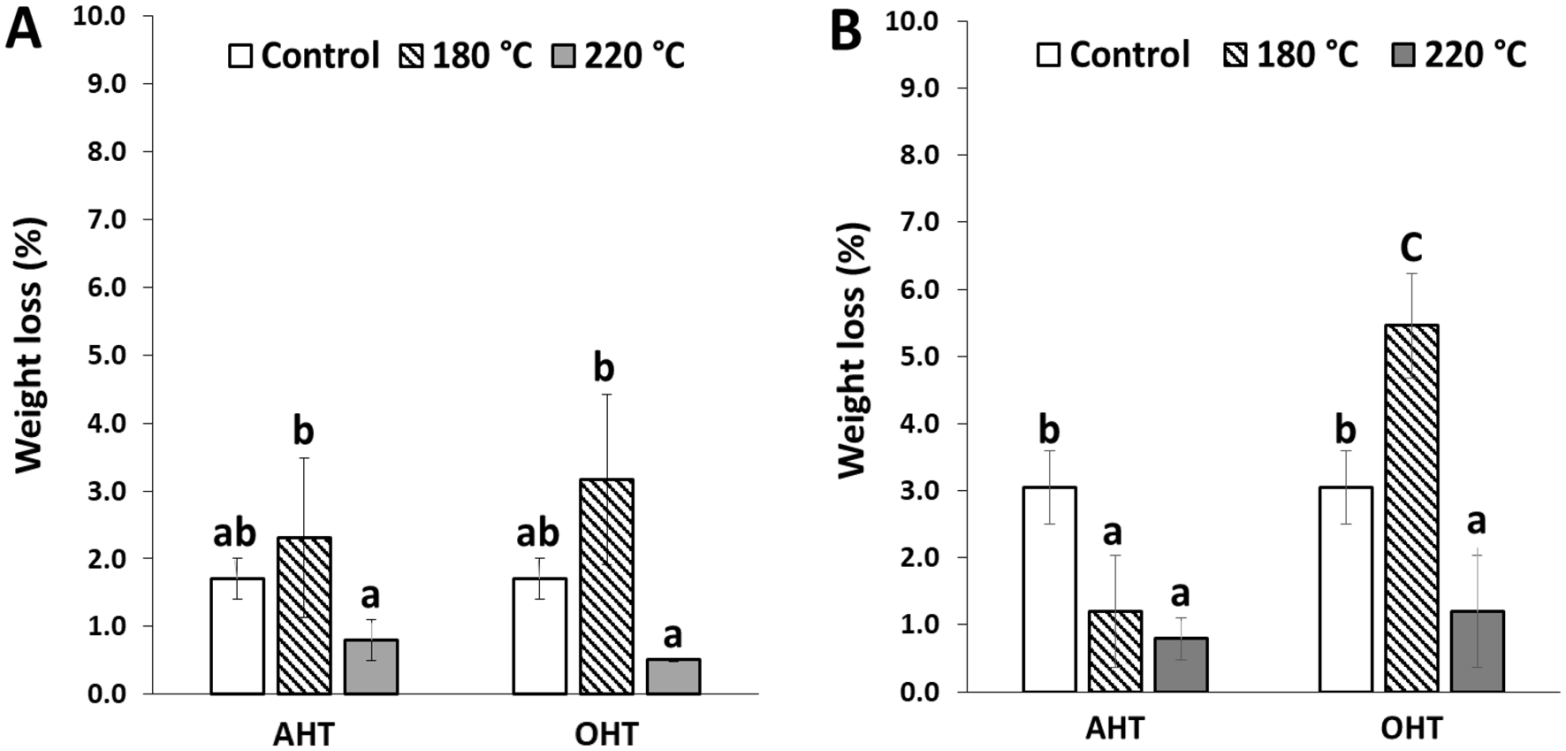
Weight loss of the untreated and treated *P. tomentosa* (**A**) and *P. koraiensis* woods (**B**) after exposure to white-rot fungus (*Trametes versicolor*). The same lowercase letters above the mean values in the histograms denote insignificant outcomes between the samples at a 5% level of significance based on Duncan’s multiple range test.

Figure 2 shows the weight loss of *P. tomentosa* and *P. koraiensis* woods heat-treated in oil and air after exposure to white-rot fungus (*Trametes versicolor*).

In *P. tomentosa* wood, the weight loss was 1.7% for untreated wood, 3.2% for OHT wood at 180 °C, and 2.3% for AHT wood at 180 °C. Furthermore, the weight loss of OHT and AHT samples at 220 °C was 0.5% and 0.8%, respectively. The weight loss was significantly higher in the heat-treated woods at 180 °C than the untreated wood and significantly decreased in the heat-treated woods at 220 °C.

In *P. koraiensis,* the weight loss of the untreated wood was 3.17%, whereas that of OHT samples at 180 °C was 5.45%, showing significantly higher weight loss in OHT samples compared to untreated wood. Furthermore, OHT samples at 220 °C had significantly lower weight loss than the untreated wood and OHT samples at 180 °C. In the AHT samples, the weight loss was significantly lower than that of the untreated wood, and there was no significant difference in weight loss between the two temperatures.

All heat-treated and untreated samples of both species exposed to white-rot fungus were highly resistant.

In the present study, heat treatment at 180 °C could not improve resistance against brown- and white-rot fungi in both wood species. In contrast, the OHT and AHT wood at 220 °C showed a higher durability against fungal attack, indicating a significant decrease in weight loss. Some previous studies on the effect of the heat treatment temperature support our results. Sailer et al.^16^ revealed that *Pinus sylvestris* and *Picea abies* woods heat-treated with linseed oil at 190–220 °C showed a significant improvement in durability against brown-rot fungus (*Coniophora puteana*). Mazela et al.^29^ reported that AHT *Pinus sylvestris* wood above 200 °C showed a significant improvement in fungal resistance against brown- (*Poria placenta*) and white-rot (*Coriolus versicolor*) fungi, whereas that below 200 °C did not show a significant change in fungal resistance. Hakkou et al.^28^ reported that AHT *Fagus sylvatica* wood at temperatures above 220 °C substantially decreased weight loss after *Coriolus versicolor* fungus exposure, showing an improvement in durability. Paul et al.^30^ reported that *Pinus sylvestris* wood that was air-heat-treated at 180 and 200 °C showed no effect on decay resistance against *Poria placenta* fungus*,* whereas a significant improvement in durability was shown in samples heat-treated at 220 and 240 °C. Moreover, the weight loss caused by brown-rot fungus (*Oilgoporus placenta*) of linseed OHT *Pinus radiata* wood at 180 and 210 °C moderately decreased^11^. Improvement in fungal durability by oil- and air-heat-treated woods at temperatures above 220 °C can be explained by the hemicelluloses being severely degraded to less hygroscopic substances, such as furfural polymers, which might form toxic compounds affecting fungal resistance^11,29,30,33^.

### Morphology

**Figure 3:**
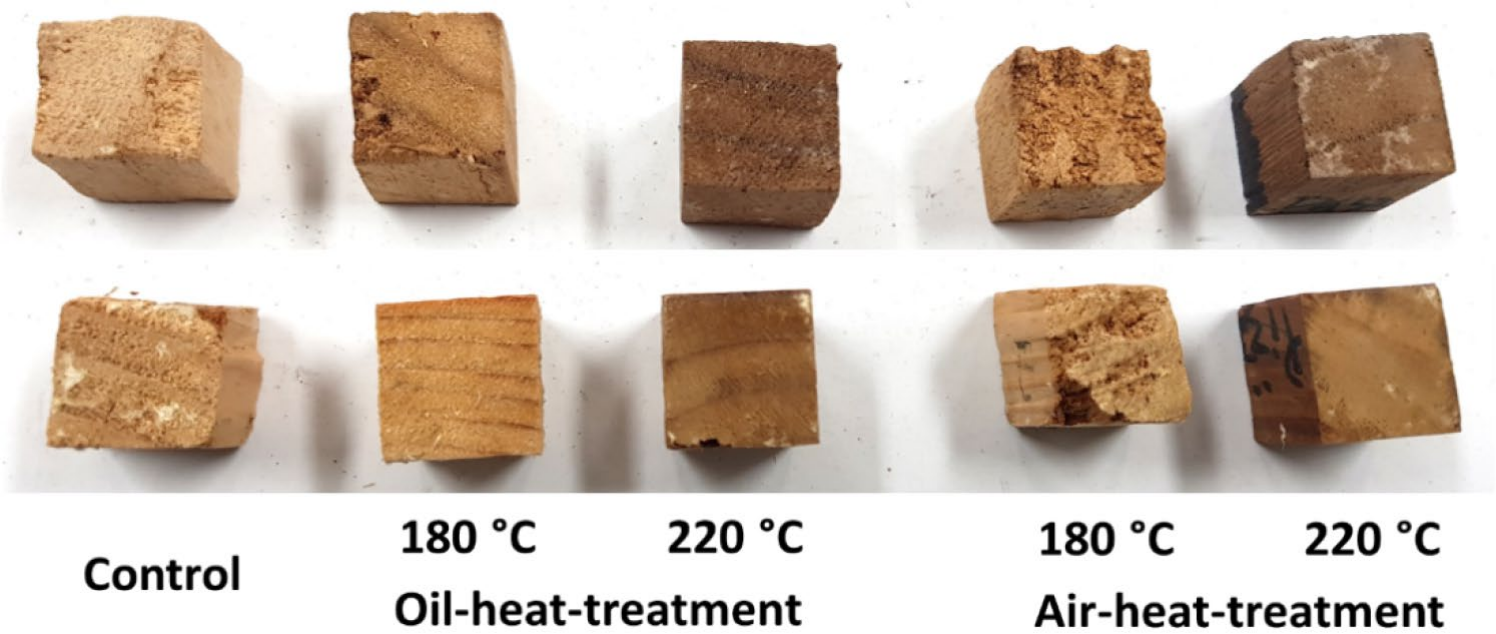
Appearances of oil- and air-heat-treated *P. tomentosa* (top row) and *P. koraiensis* (bottom row) wood after exposure to brown-rot fungus.

**Figure 4:**
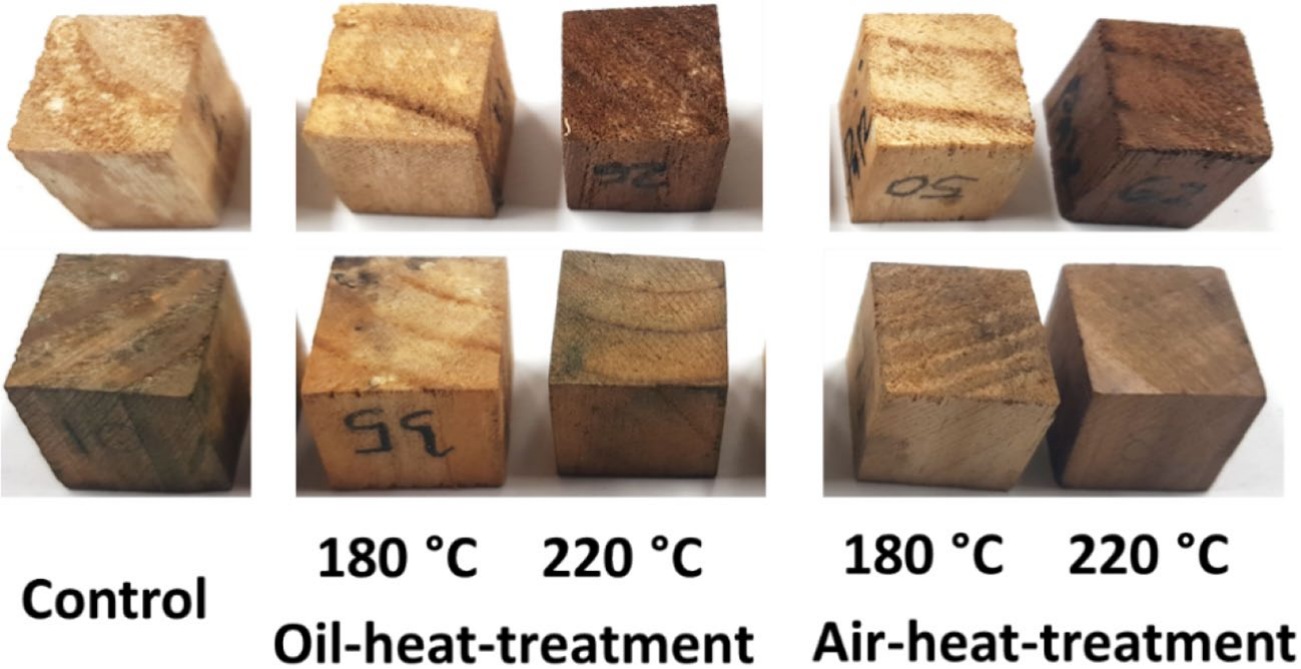
Appearances of oil- and air-heat-treated *P. tomentosa* (top row) and *P. koraiensis* (bottom row) after exposure to white-rot fungus.

The appearances of OHT and AHT *P. tomentosa* and *P. koraiensis* woods after exposure to brown- (*Fomitopsis palustris*) and white-rot (*Trametes versicolor*) fungi are shown in Figs. 3 and 4, respectively. The AHT samples at a temperature of 180 °C showed severe damage on the transverse surface after brown-rot fungus exposure, whereas the other samples retained their shape. In contrast, after white-rot fungus exposure, all the samples retained their shape.

**Figure 5:**
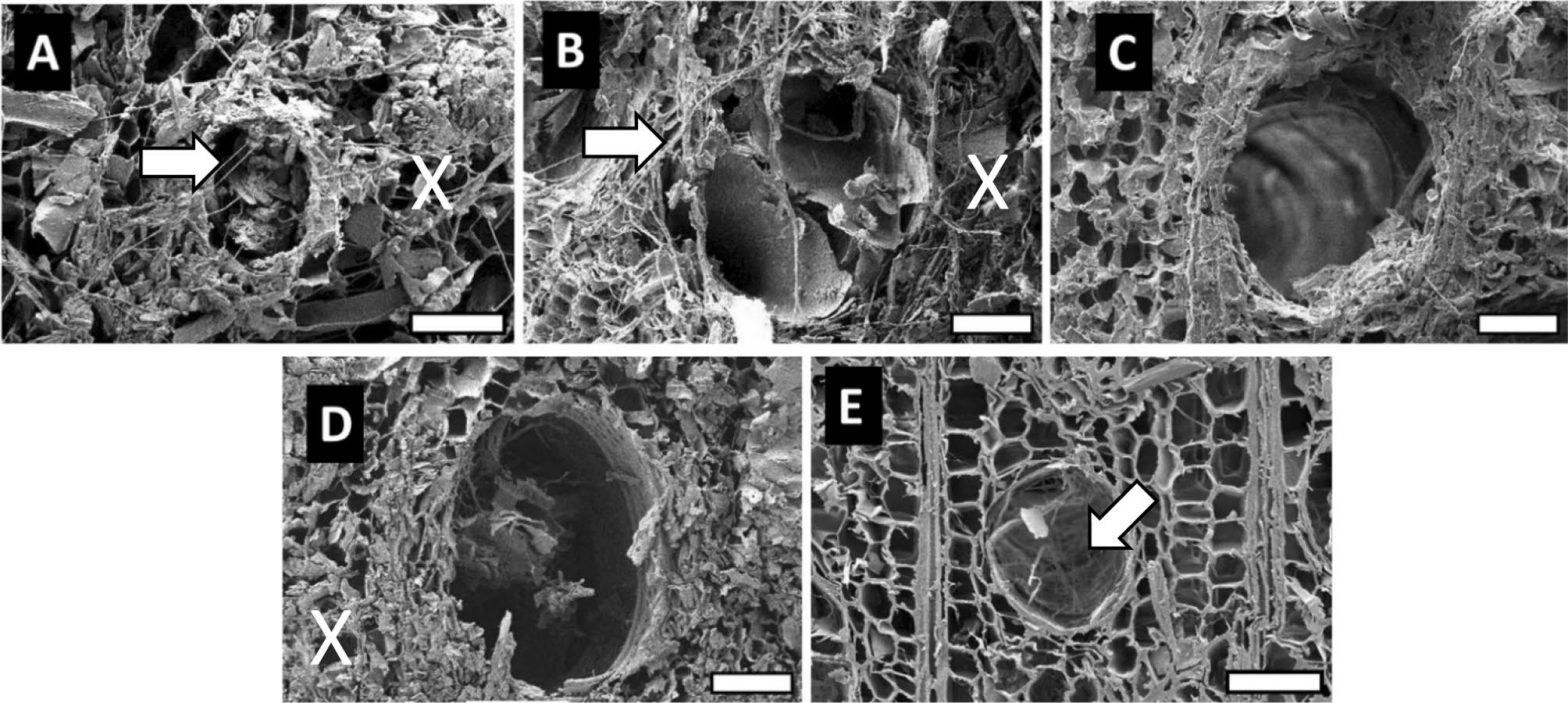
Transverse surface of *P. tomentosa* wood exposed to the brown-rot fungus. (**A**) untreated wood; (**B**,**C**) AHT wood at 180 and 220 °C; (**D**,**E**) OHT wood at 180 and 220 °C. Hyphae (arrow) and damaged parenchyma cells (X). Scale bars 75 µm.

**Figure 6:**
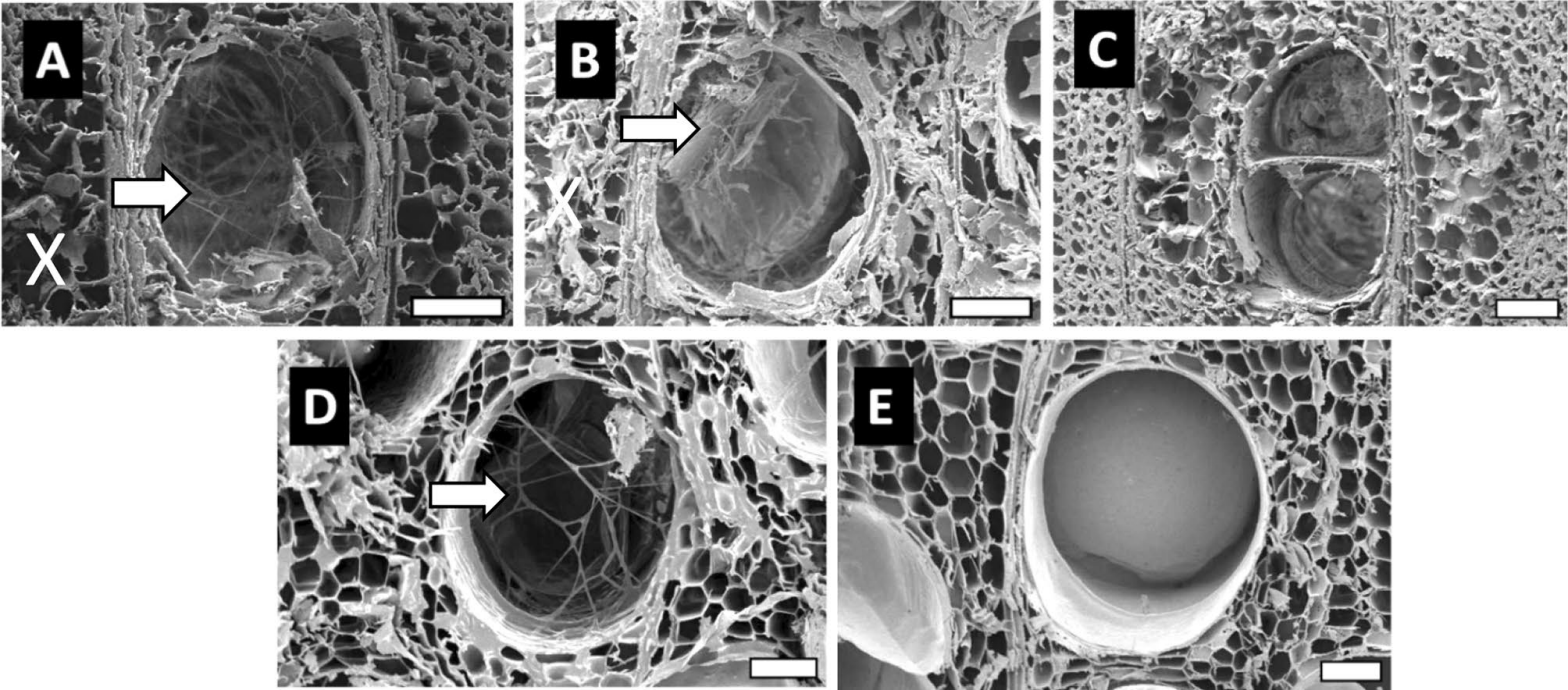
Transverse surface of *P. tomentosa* wood exposed to the white-rot fungus. (**A**) Untreated wood; (**B**,**C**) AHT wood at 180 and 220 °C; (**D**,**E**) OHT wood at 180 and 220 °C. Hyphae (arrow) and damaged parenchyma cells (X). Scale bars 75 µm.

Scanning electron micrographs of the transverse surfaces of *P. tomentosa* exposed to brown- and white-rot fungi are presented in Figs. 5 and 6, respectively.

In brown-rotted *P. tomentosa* wood, the untreated and heat-treated samples at 180 °C showed severely damaged parenchyma cells, whereas the vessels retained their lumen shape. In white-rotted *P. tomentosa*, hyphae were observed in the vessel lumen of untreated and heat-treated woods at 180 °C, whereas the hyphae were scarcely observed in the parenchyma cell.

In heat-treated wood at 220 °C, hyphae were observed in the vessel lumen of OHT wood attacked by both fungi, whereas hyphae were scarcely observed in those of AHT wood. Moreover, most cells were not damaged by the fungi.

**Figure 7:**
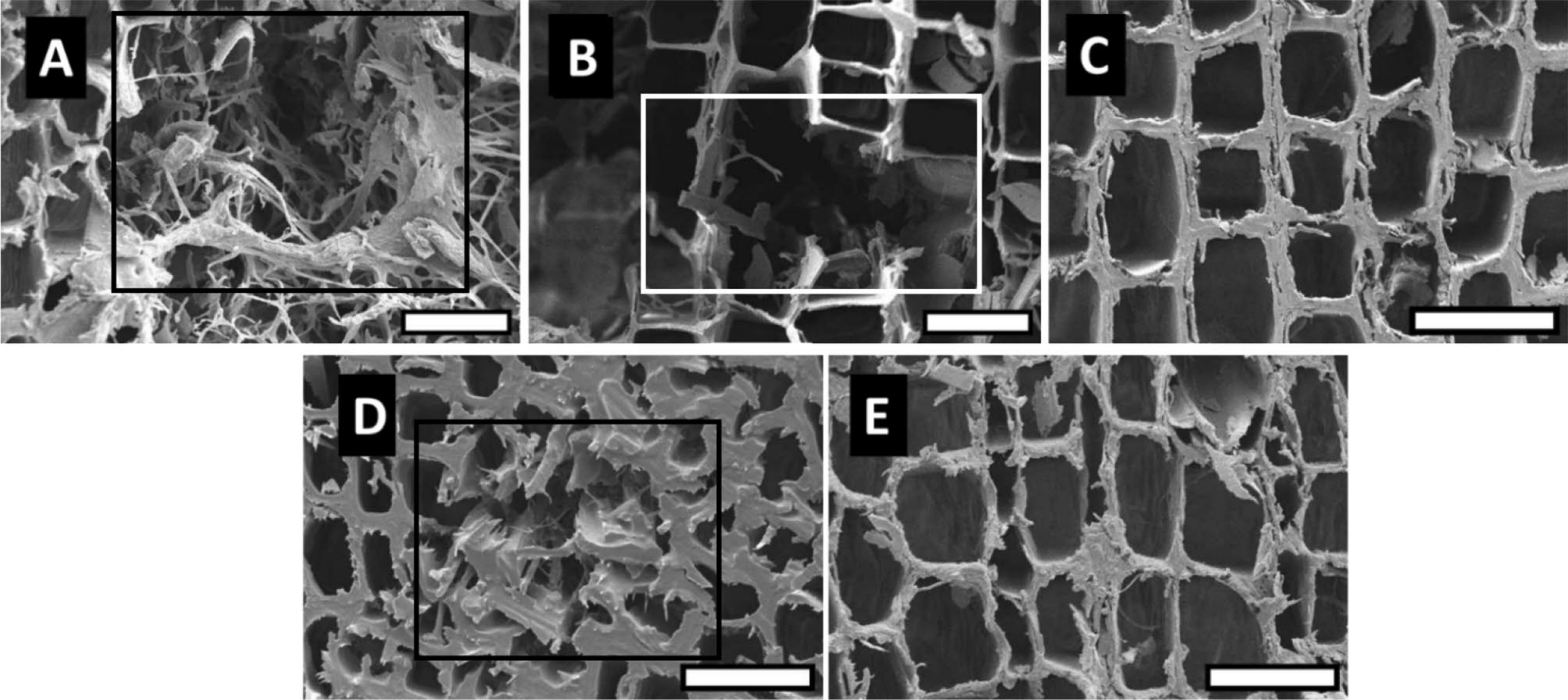
Transverse surface of *P. koraiensis* wood exposed to the brown-rot fungus. (**A**) Untreated wood; (**B**,**C**) AHT wood at 180 and 220 °C; (**D**,**E**) OHT wood at 180 and 220 °C. Collapsed tracheid (rectangular). Scale bars 35 µm.

**Figure 8:**
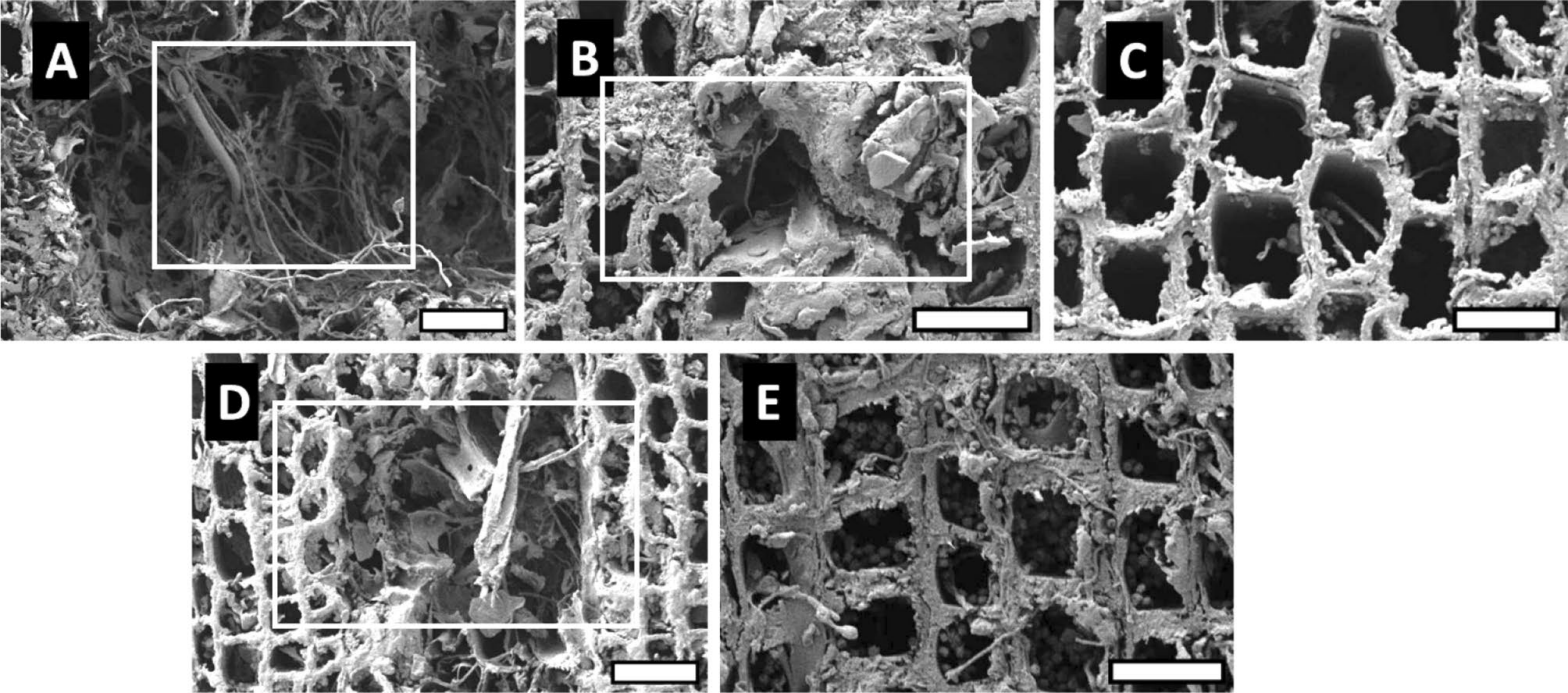
Transverse surface of *P. koraiensis* wood exposed to the white-rot fungus. (**A**) Untreated wood; (**B**,**C**) AHT wood at 180 and 220 °C; (**D**,**E**) OHT wood at 180 and 220 °C. Collapsed tracheid (rectangular). Scale bars 35 µm.

Scanning electron micrographs of the transverse surfaces of *P. koraiensis* wood exposed to brown- and white-rot fungi are presented in Figs. 7 and 8, respectively. In brown- and white-rotted wood, the untreated and heat-treated samples at 180 °C showed severely collapsed tracheids. In heat-treated wood at 220 °C, hyphae were scarcely observed in the tracheid, and collapsed cells were absent.

Some studies support the results of the present study on the morphology of brown- and white-rotted woods. Wilcox et al.^34^ reported that *Abies concolor* exposed to the brown-rot fungus in the latewood and earlywood showed cell separation. Anagnost^35^ reported that, in the transverse section, the earlywood of brown-rotted *Pinus taeda* and *Pseudotsuga menziesii* showed collapsed cells and transwall fractures due to cell weakening. Lee et al.^36^ reported that a brown-rot fungus (*Coniophora puteana*) degraded the tracheids of *Pinus densiflora* and fibers of *Quercus acutissima*, whereas the vessel walls retained their shape.

Blanchette et al.^37^ reported that degradation by various white-rot *Basidiomycetes* caused the loss of fibers, tracheids, and parenchyma cells, but not vessels. The large vessel elements remained intact after the other cells were completely degraded. Akhtar et al.^38^ reported that white-rot fungi enter the cell lumen and rapidly colonize ray parenchyma cells that contain free sugars and other nutrients. White-rot fungi that simultaneously attack all cell wall components caused a localized erosion of all cell wall layers. Anagnost^35^ reported that erosion channels were observed in the transverse section of white-rotted birch (*Exidia glandulosa* wood as localized notches extending from the fiber lumen to the middle lamella; degraded cell walls were also present in some areas. In addition, cell separation in the transverse section was observed in birch wood attacked by a white-rot fungus (*Mycena leaiana*).

### Crystalline properties

**Figure 9:**
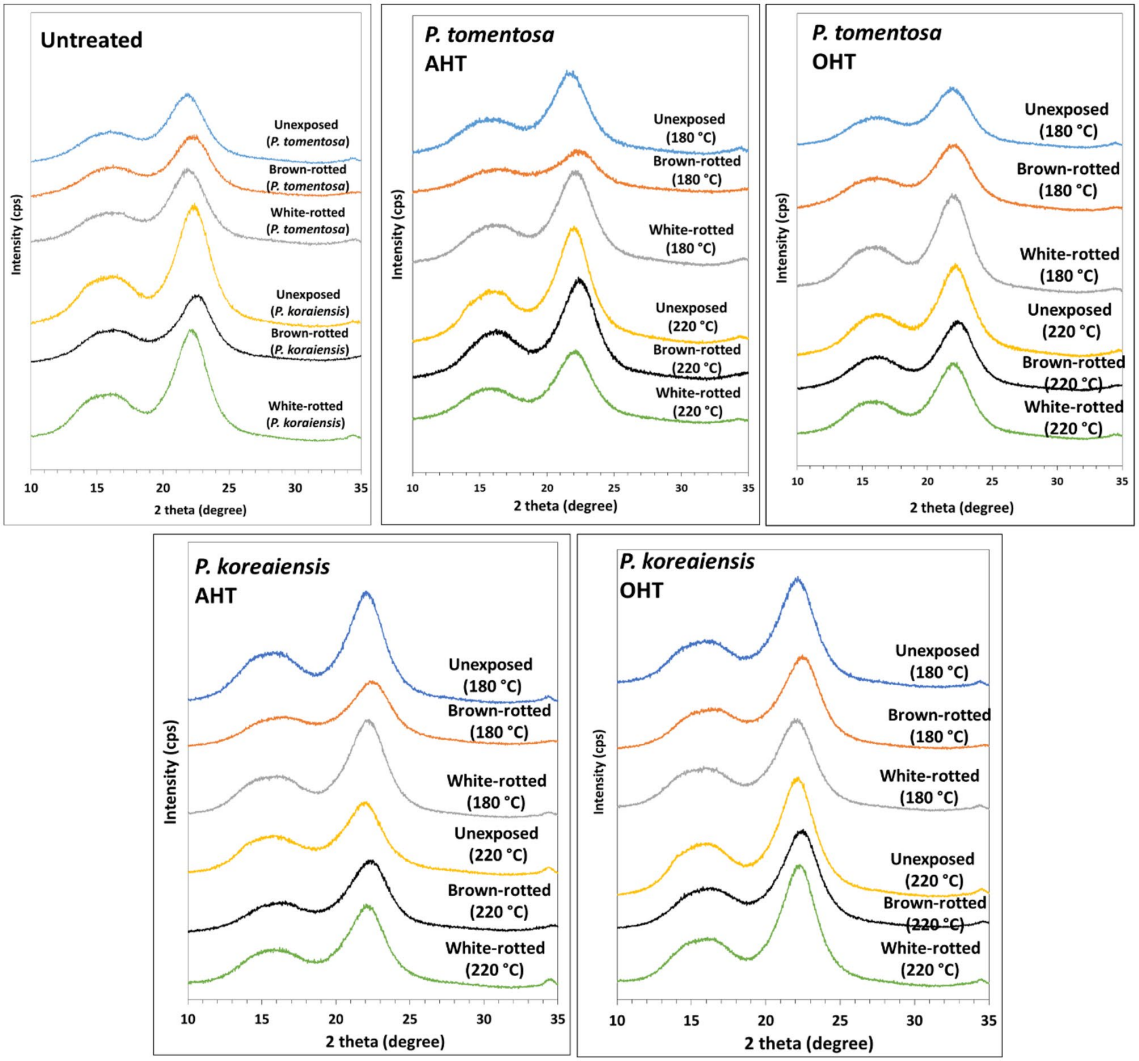
X-ray diffraction patterns before and after the fungi exposure of untreated, AHT, and OHT*P. Tomentosa* and *P. koraiensis* woods.

The X-ray diffractograms of untreated and heat-treated *P. tomentosa* and *P. koraiensis* woods before and after fungal exposure are presented in Fig. 9. The diffractograms of each sample showed a cellulose I crystalline pattern.

The relative crystallinity (RC) of the untreated and heat-treated *P. tomentosa* wood after exposure to brown- and white-rot fungi are shown in Table 4. Brown-rot fungus exposure decreased the RC of untreated *P. koraiensis* wood and both AHT woods at 180 °C, while the untreated and heat-treated woods at 180 °C exposed to white-rot fungus showed higher RC than the brown-rotted wood. The heat-treated wood at 220 °C showed no notable change in RC before and after exposure to both fungi, indicating higher durability against fungi.

**Table 4:** Relative crystallinity of untreated and heat-treated wood before and after exposure to fungi.

		Relative crystallinity (%)		
		Unexposed	Brown-rotted	White-rotted
*P. tomentosa*	Control	55.0 ± 3.2	55.0 ± 1.3	59.2 ± 1.3
	AHT at 180 °C	57.8 ± 3.2	49.3 ± 2.6	59.0 ± 1.0
	AHT at 220 °C	62.1 ± 3.6	63.4 ± 5.0	61.4 ± 1.5
	OHT at 180 °C	56.7 ± 2.1	52.1 ± 3.1	63.1 ± 1.3
	OHT at 220 °C	59.4 ± 3.3	58.8 ± 3.9	62.2 ± 1.9
*P. koraiensis*	Control	69.5 ± 2.9	48.7 ± 5.7	55.3 ± 4.6
	AHT at 180 °C	67.6 ± 4.1	57.7 ± 3.3	66.2 ± 1.9
	AHT at 220 °C	65.1 ± 2.5	63.0 ± 3.3	67.1 ± 3.5
	OHT at 180 °C	64.9 ± 3.5	62.3 ± 2.1	64.7 ± 5.2
	OHT at 220 °C	68.4 ± 1.7	66.6 ± 3.2	69.3 ± 2.2

The RC of *P. tomentosa* wood unexposed to fungi significantly increased after heat treatment at 220 °C. In brown-rotted wood, the RC of AHT and OHT *P. tomentosa* wood showed a notable increase at 220 °C, whereas white-rotted *P. tomentosa* wood showed a slight increase in RC after heat treatment. In unexposed *P. koraiensis* wood, the RC was comparable between untreated and heat-treated wood. The RC of brown- and white-rotted *P. koraiensis* wood showed a noticeable increase after heat treatment.

Several studies support our results regarding variations in RC caused by fungi. Howell et al.^39^ reported that the RC of pine wood exposed to white-rot fungus (*Serpula lacrymans*) for eight weeks was higher than that of the unexposed pine wood, whereas the wood exposed to brown-rot fungus (*Gloeophyllum trabeum)* for eight weeks showed lower RC than the unexposed samples. Hastrup et al.^40^ reported that the RC of red maple substantially decreased after exposure to *Meruliporia incrassata* (brown-rot fungus), whereas wood exposed to *Gloeophyllum trabeum* (brown-rot fungus) showed a constant RC until nine weeks of exposure and severely decreased at twelve weeks of exposure. In addition, the RC of wood decayed by white-rot fungi (*Irpex lacteus* and *Pycnoporus sanguineus*) increased at nine weeks of exposure. The increase in RC may be related to the removal of hemicelluloses and easily available non-crystalline cellulose during decay^39,40^. In addition, the decrease in RC could be due to fungal attack on crystalline cellulose after the degradation of the more readily available amorphous nutrient sources. Heat treatment above 200 °C substantially degraded hemicelluloses and reduced the hygroscopicity of wood, preventing the diffusion of degradation products of carbohydrates to the fungal hyphae^41^. In addition, severe degradation of hemicelluloses may result in the formation of toxic compounds that can improve fungal resistance^11,29,30,33^.

The CW of the untreated and heat-treated wood before and after fungal exposure are listed in Table 5. In untreated and heat-treated *P. tomentosa* wood, the CW before and after brown- and white-rot fungal exposure showed similar values. Furthermore, there was no difference in CW with increasing temperature.

**Table 5:** Crystallite width of untreated and heat-treated wood before and after fungal exposure.

		Crystallite width (nm)		
		Unexposed	Brown-rotted	White-rotted
*P. tomentosa*	Control	2.8 ± 0.1	2.7 ± 0.1	2.6 ± 0.1
	AHT at 180 °C	2.7 ± 0.1	2.6 ± 0.1	2.7 ± 0.1
	AHT at 220 °C	2.9 ± 0.2	2.7 ± 0.1	2.7 ± 0.1
	OHT at 180 °C	2.7 ± 0.2	2.5 ± 0.1	2.8 ± 0.1
	OHT at 220 °C	2.7 ± 0.1	2.7 ± 0.1	2.7 ± 0.1
*P. koraiensis*	Control	2.9 ± 0.2	2.4 ± 0.3	2.5 ± 0.0
	AHT at 180 °C	2.8 ± 0.1	2.6 ± 0.1	2.8 ± 0.1
	AHT at 220 °C	2.8 ± 0.0	2.8 ± 0.1	2.8 ± 0.1
	OHT at 180 °C	2.9 ± 0.2	2.9 ± 0.1	2.8 ± 0.1
	OHT at 220 °C	3.1 ± 0.1	3.0 ± 0.1	3.0 ± 0.1

In *P. koraiensis* wood, the CW of untreated wood before fungal exposure was slightly higher than that of after fungal exposure, whereas the CW was comparable between brown- and white-rotted wood. Additionally, the CW of AHT and OHT *P. koraiensis* wood before and after brown- and white-rot exposure were comparable. The CW of the heat-treated wood after fungal exposure was slightly higher than that of the untreated wood. No fungal effect on the CW of *P. koraiensis* wood was detected in heat-treated wood, which could be related to an increase in fungal durability. Heat treatment above 200 °C severely degraded hemicelluloses, reducing the hygroscopic properties of the wood, which can prevent or limit fungal growth. Moreover, severe degradation of hemicellulose into furfural polymers may form toxic compounds that limit fungal growth^11,29,30,33,41^.

#### Chemical compound analysis

**Figure 10:**
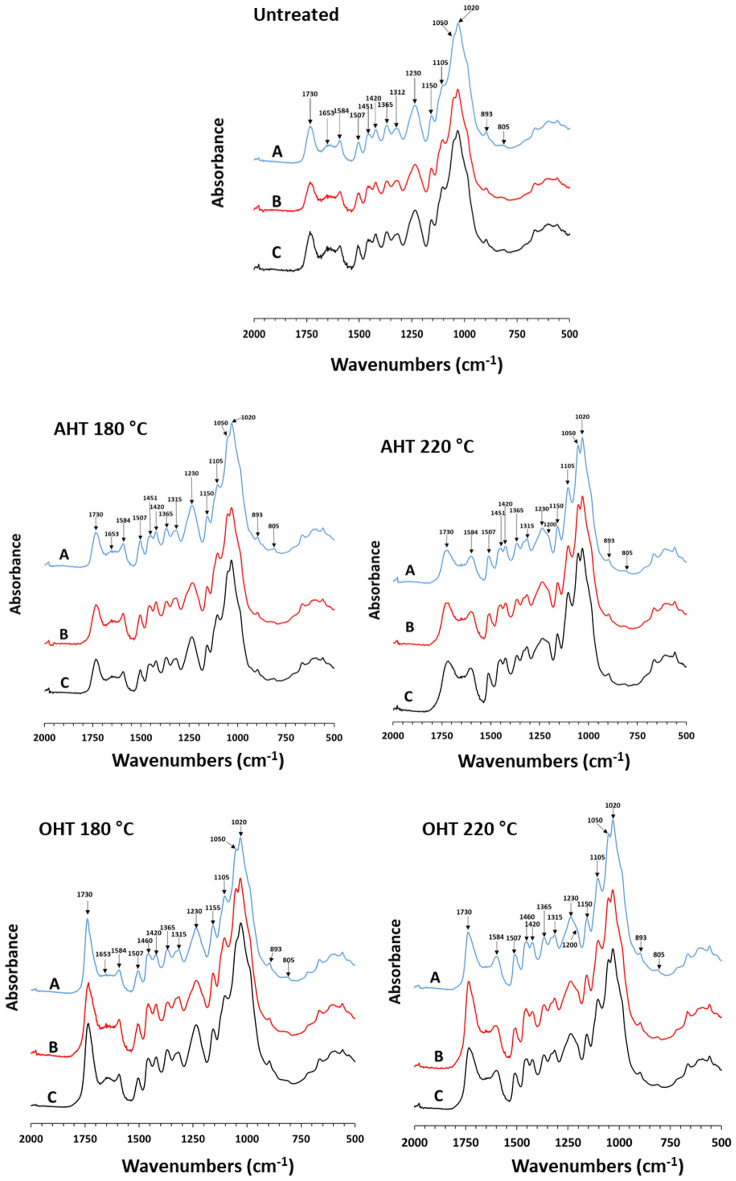
FTIR spectra of untreated and heat-treated *P. tomentosa* wood before (**A**) and after brown- (**B**) and white-rot (**C**) fungal exposure.

**Figure 11:**
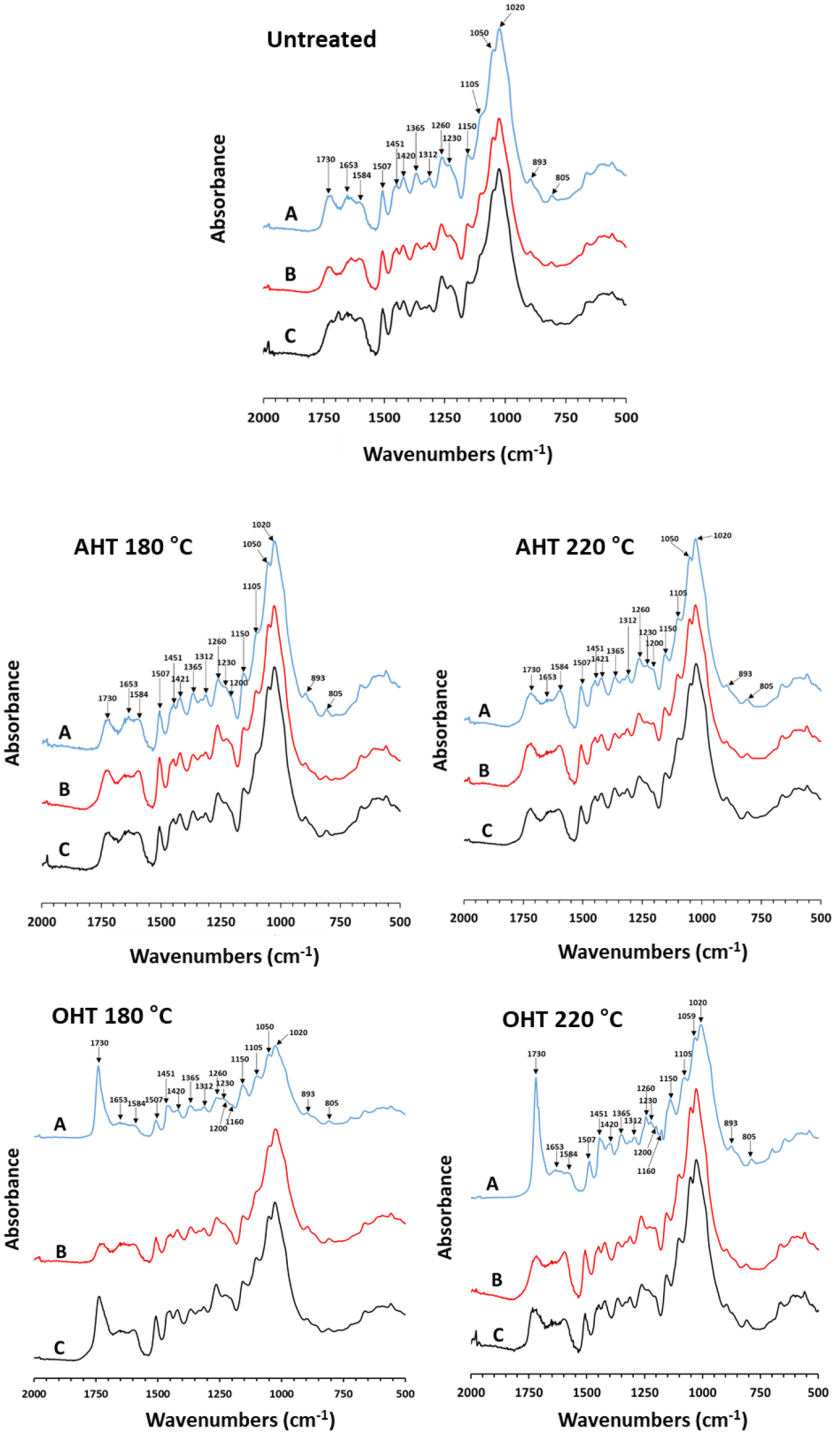
FTIR spectra of *P. koreaiensis* before (**A**) and after brown- and white-rot fungal exposure (**B**,**C**, respectively).

The FTIR spectra of *P. tomentosa* and *P. koreaiensis* woods before and after fungal exposure are shown in Figs. 10 and 11, respectively.

#### Unexposed wood to fungi

The untreated *P. tomentosa* and *P. koreaiensis* woods showed peaks at 1738 cm^−1^ wavenumber for the C=O stretch (unconjugated) in esters, ketones, aldehydes, and acids, and at 1653 cm^−1^ wavenumber, representing absorbed O–H and conjugated C–O in polysaccharides. Aromatic skeletal vibrations in the lignin ofuntreated wood of both species were detected at 1584, 1507, 1451, and 1420 cm^−1^ wavenumbers. In both the untreated woods, C–H deformation in cellulose and hemicellulose CH_2_ wagging in crystalline cellulose were detected at 1365 and 1312 cm^−1^ wavenumbers, respectively. The peaks at 1260 cm^−1^ wavenumber corresponding to C–O vibrations in guaiacyl rings and at 1230 cm^−1^ wavenumber representing OH vibrations in guaiacyl rings, C–C, C–O, and C=O stretches in lignin, were detected in *P. koreaiensis*. The peak at 1230 cm^−1^ wavenumber was detected in *P. tomentosa*, whereas the peak at 1260 cm^−1^ wavenumber was absent in *P. tomentosa*.

A shoulder peak at 1200 cm^−1^ wavenumber representing C–O stretching in cellulose occurred in untreated *P. koriaensis*, whereas this peak was absent in *P. tomentosa*. The peaks at 1150, 1105, 1051, 1020, and 893 cm^−1^ wavenumbers correspond to the aromatic C–H in-plane deformation, secondary alcohols, C=O stretching, C–O–C stretching in cellulose and hemicellulose, C–O stretching of secondary alcohols, C–O stretching in primary alcohols in cellulose, and the anomeric carbon group frequency in cellulose and hemicellulose, respectively, which were detected in both species. Vibrations of mannan in hemicellulose and CH out-of-plane bending in phenyl rings were detected at 805 cm^−1^ wavenumber.

In AHT *P. tomentosa* and *P. koreaiensis* woods, the peak intensity at 1653 cm^−1^ wavenumber decreased with increasing temperature, whereas the peak intensities at 1050 and 1105 cm^−1^ wavenumber increased with increasing temperature. The peak at 1230 cm^−1^ wavenumber for lignin in both AHT woods at 220 °C decreased. The shoulder peak at 1200 cm^−1^ wavenumber of C–O stretching in cellulose was detected in AHT *P. tomentosa* wood at 220 °C and became notable in AHT *P. koreaiensis* wood at 220 °C.

In the OHT of both woods, the peak at 1730 cm^−1^ wavenumber was noticeably higher than that of the untreated and AHT woods. The peaks at 1105 and 1050 cm^−1^ wavenumbers for carbohydrates and 1420 cm^−1^ wavenumber for lignin increased with oil-heat-treatment. In the OHT *P. tomentosa* wood, the peak at 1653 cm^−1^ wavenumber was absent, and a shoulder peak at 1200 cm^−1^ wavenumber of carbohydrates was detected. In the OHT *P. koraiensis* wood, the peaks at 1653 and 1584 cm^−1^ wavenumbers decreased. The peaks at 1260, 1230, and 1200 cm^−1^ wavenumbers decreased in OHT *P. koraiensis* wood at 180 °C, whereas the peaks increasedin OHT *P. koraiensis* wood at 220 °C. The peak at 1160 cm^−1^ wavenumbers representing C–O–C antisymmetric bridge stretching vibration of carbohydrates was detected in OHT *P. koraiensis* wood at 220 °C.

##### Brown-rotted wood

In the present study, the peaks at 1730, 1365, and 893 cm^−1^ wavenumbers representing carbohydrates of untreated and heat-treated *P. tomentosa* wood at 180 °C decreased after brown-rot exposure. The peaks at 1105 and 1050 cm^−1^ wavenumbers of carbohydrates in untreated and AHT *P. tomentosa* wood at 180 °C increased after brown-rot exposure. In AHT wood at 220 °C, no noticeable change in the spectra was observed after brown-rot exposure. In OHT *P. tomentosa* wood at 180 °C, the peaks at 1315 and 1050 cm^−1^ wavenumbers of carbohydrates increased after brown-rot exposure, whereas in OHT samples at 220 °C, the peak at 1315 cm^−1^ wavenumber of carbohydrates increased after brown-rot exposure.

In untreated *P. koreaiensis* woods, after brown-rot fungus exposure, the carbohydrate peaks at 1730, 1365, 893, and 805 cm^−1^ wavenumbers decreased, whereas the carbohydrate peaks at 1105 and 1050 cm^−1^ increased. The carbohydrate peaks of AHT *P. koreaiensis* wood at 180 °C, such as 1653, 1365, 1230, 1150, 893, and 805 cm^−1^ wavenumbers, decreased in intensity after brown-rot fungi exposure, whereas the peak at 1105 cm^−1^ wavenumbers increased after brown-rot fungus exposure. In AHT samples at 220 °C, the spectra showed no noticeable changes after brown-rot fungus exposure. The peak intensities at 1730, 1365, 1150, and 1105 cm^−1^ wavenumbers representing carbohydrates in OHT *P. koreaiensis* wood at 180 and 220 °C decreased after brown-rot fungus exposure, whereas the peak at 1160 cm^−1^ wavenumber representing carbohydrates was absent after brown-rot fungus exposure.

In untreated and heat-treated *P. tomentosa*, the peaks of the aromatic skeletal vibrations in lignin at 1584, 1507, 1451, and 1420 cm^−1^ wavenumbers had similar intensities before and after fungal exposure. In untreated *P. koreaiensis,* the lignin peak at 1420 cm^−1^ wavenumbers decreased after fungus exposure.

In the AHT *P. koreaiensis* wood, the lignin peaks showed no noticeable changes after exposure to brown-rot fungus. In untreated and OHT *P. koreaiensis* woods, 1420 and 1230 cm^−1^ wavenumbers of lignin decreased after exposure to brown-rot fungus. In addition, the lignin peak at 1584 cm^−1^ wavenumber in the untreated wood increased in intensity after fungal exposure.

In the present study, the noticeable change in FTIR spectra of polysaccharides occurred at 1730, 1365, 893, and 805 cm^−1^ wavenumbers, which aligned with the results of Pandey and Pitman^42^, Pandey and Nagveni^43^, Li et al.^44^, and Yilgor et al.^45^. As they described, a severe decrease in polysaccharide bands at 1738, 1375, 1158, and 898 cm^−1^ wavenumbers occurred in wood decayed by brown-rot fungi. In the present study, 1105 and 1050 cm^−1^ wavenumbers of untreated wood and AHT 180 °C wood of both species increased after brown-rot exposure. Yilgor et al.^45^ reported that a shoulder peak at 1106 cm^−1^ wavenumber of O–H association band in cellulose and hemicelluloses increased substantially after brown-rot fungi (*T. palustris* and *G. trabeum*) exposure due to its response to deuteration in cellulose, which supports our results.

##### White-rotted wood

Exposure to white-rot fungus did not cause noticeable changes inthe lignin peaks of untreated and heat-treated *Paulownia* wood, whereas the peaks at 1105 and 1050 cm^−1^ wavenumbers of carbohydrates increased after white-rot fungi exposure.

In the untreated *P. koraeinsis* wood, the peaks of carbohydrates (1150, 1105, and 1050 cm^−1^ wavenumbers) and lignin (1420 and 1315 cm^−1^ wavenumbers) showed lower intensities after white-rot fungus exposure. The FTIR spectra of AHT wood at 180 and 220 °C did not change after white-rot fungus exposure. The peak intensity at 1730 and 1365 cm^−1^ wavenumbers of carbohydrates and 1451 and 1230 cm^−1^ wavenumbers of lignin in OHT woods at 180 °C decreased after white-rot fungus exposure. In OHT samples at 220 °C, the peaks at 1730, 1200, 1160, and 1150 cm^−1^ wavenumbers of carbohydrates and 1451 and 1230 cm^−1^ wavenumbers of lignin decreased after fungus exposure.

As reported by Faix et al.^46^, in beech wood decayed by *T. versicolor*, the bands between 1200 and 900 cm^−1^ wavenumbers primarily due to polysaccharides and hemicellulose bands at 1740 cm^−1^ wavenumber showed minor change. The attack of *T. versicolor* on wood noticeably increased the intensity of the peak at 1646 cm^−1^ wavenumber from lignin and decreased the peak intensities at 1596 and 1506 cm^−1^ wavenumbers corresponding to aromatic skeletal vibrations, suggesting a structural change and loss of aromatic units during fungal degradation. Bari et al.^47^ reported that *Picea abies*, *Fagus orientalis*, and *Quercus castaneifolia* showed a decrease in the intensities of polysaccharides (1733, 1236, 1369, 1157, and 897 cm^−1^ wavenumbers) and lignin peaks (1594 and 1504 cm^−1^ wavenumbers).

## Conclusion

The effects of oil- and air-heat-treatment on the fungal durability of *P. tomentosa* and *P. koraiensis* were examined, and the results were as follows:

1. Heat treatment at 220 °C significantly decreased weight loss due to brown- and white-rot fungal exposure in both species. Parenchyma cells in *P. tomentosa* and tracheids in *P. koraiensis* from the untreated and heat-treated wood at 180 °C were severely damaged by fungal exposure.
2. Brown-rot fungus exposure decreased the RC of untreated *P. koraiensis* wood and AHT wood of both species at 180 °C. In untreated and heat-treated woods at 180 °C, the RC of brown-rotted wood was lower than that of white-rotted woods. In addition, fungal exposure did not affect the RC in heat-treated wood at 220 °C. The crystallite widths of the untreated and heat-treated *P. tomentosa* and *P. koraiensis* woods showed no considerable changes before and after fungal exposure.
3. After brown-rot fungus exposure, untreated and heat-treated wood at 180 °C showed a noticeable change in polysaccharide peaks, whereas no noticeable change in lignin peaks was observed. Heat-treated wood at 220 °C of both species showed no noticeable change in the FTIR spectra after brown-rot fungus exposure. No noticeable change in the FTIR spectra of the wood was observed after exposure to the white-rot fungus.

In conclusion, AHT and OHT at 220 °C significantly improved the durability of *P. tomentosa* and *P. koraiensis* wood against brown- and white-rot fungi. The results of the present study can be used to understand the fungal durability of heat-treated wood for the effective utilization of wood from both species.

## Data Availability

The datasets generated and analyzed during the current study are not publicly available but are available from the corresponding author on reasonable request.
